# A simple, efficient and rapid HPLC–UV method for the detection of 5-HT in RIN-14B cell extract and cell culture medium

**DOI:** 10.1186/s13065-019-0591-x

**Published:** 2019-06-12

**Authors:** Qiangqiang He, Maoru Li, Xuechun Wang, Zhenjiang Xia, Yuzhi Du, Yan Li, Lixin Wei, Jing Shang

**Affiliations:** 1 Qinghai Key Laboratory of Tibetan Medicine Pharmacology and Safety Evaluation, Chinese Academy of Sciences - Northwest Institute of Plateau Biology, Xining, 810008 Qinghai China; 2 Key Laboratory of Tibetan Medicine Research, Chinese Academy of Sciences - Northwest Institute of Plateau Biology, Xining, 810008 Qinghai China; 3 University of Chinese Academy of Sciences, Beijing, 100049 China; 4 State Key Laboratory of Natural Medicines, China Pharmaceutical University, Nanjing, 21198 China; 5 Jiangsu Key Laboratory of TCM Evaluation and Translational Research, China Pharmaceutical University, Nanjing, 211198 China; 6 School of Traditional Chinese Pharmacy, China Pharmaceutical University, Nanjing, 211198 China

**Keywords:** HPLC–UV, Bioanalytical method validation, Cell culture medium, 5-Hydroxytryptamine, RIN-14B cells

## Abstract

5-Hydroxytryptamine (also known as 5-HT, serotonin) is one of the monoamine neurotransmitters which is distributed widely in plasma and brain of mammals and plays important roles in physiological manipulations. In the present method, we describe the development of a simple, efficient and rapid high performance liquid chromatographic method coupled with ultraviolet (HPLC–UV) detector for the qualitative and quantitative analysis of 5-HT in both cell extract and cell culture medium (RIN-14B). The experiments use repeated freeze–thaw cycles followed by centrifugation and direct injection of the supernatant into the chromatography. An analytical C18 column (Agilent Zorbax Extend, 4.6 × 250 mm, 5 μm.) was taken for chromatographic separation; the mobile phase was 0.05 mol/L potassium dihydrogen phosphate (KH_2_PO_4_)/acetonitrile (90:10 v/v). Isocratic elution is established at the flow rate of 1.0 mL/min. The time required for this chromatographic run is 8 min. Over the concentration range of 0.1–10 μg/mL, the calibration curve is linear in this method. Other unique characteristics and advantages include high accuracy (92.02–103.28%) and high precision (intra- and inter-day coefficients of variation ≤ 4.69%). This method is applicable for the investigation of drug/condition–response relationships in the function of synthesis and secretion of 5-HT in cultured RIN-14B cells in various in vitro studies.

## Introduction

**Fig. 1:**
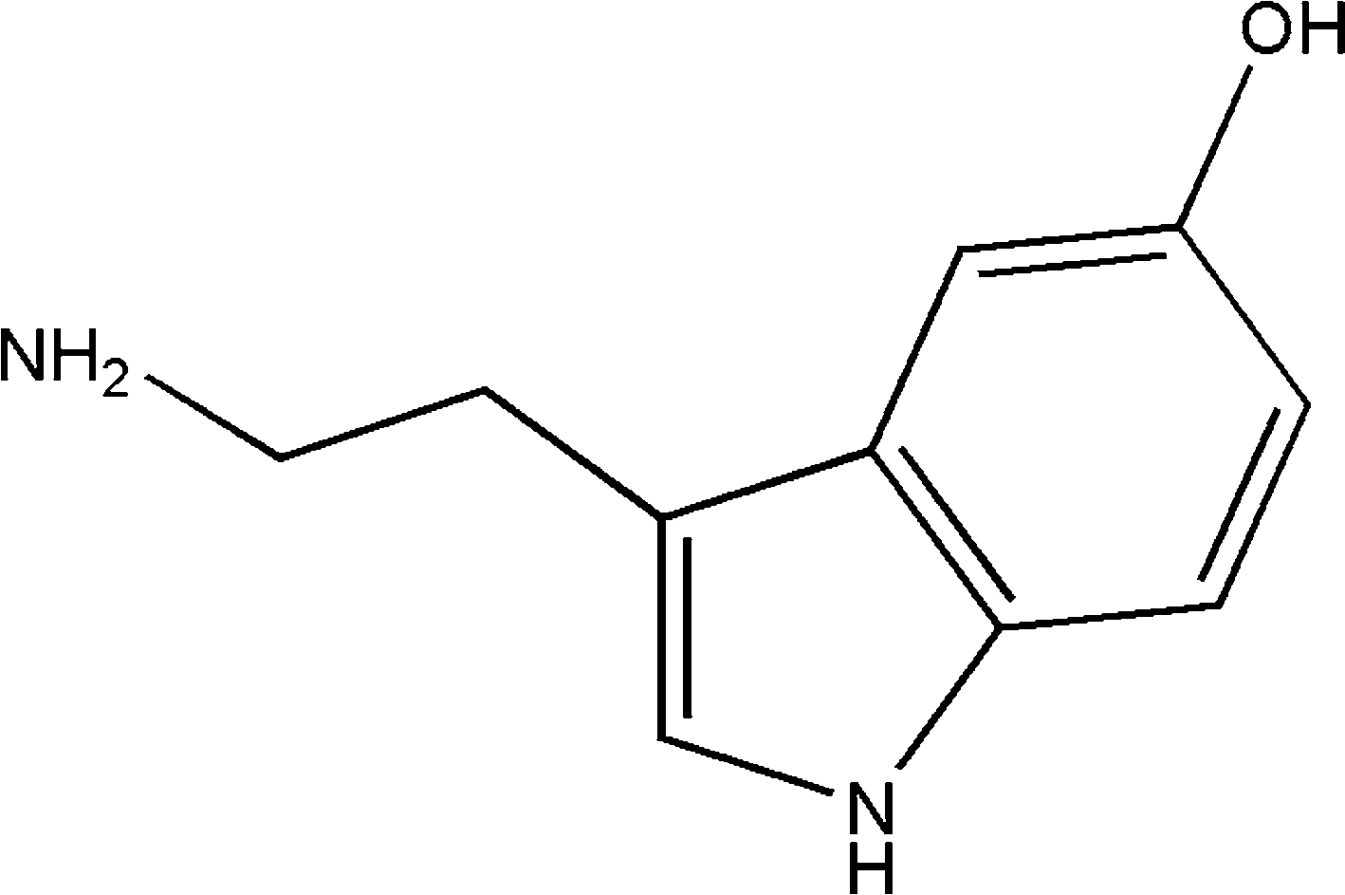
Chemical structure of 5-HT

5-Hydroxytryptamine (also known as 5-HT, serotonin, Fig. 1) which is released from the enterochromaffin (EC) cells is an important neurotransmitter in both the central and peripheral nervous systems [1]. EC cells are a sub-type of enteroendocrine (EE) cells and can be found among the enterocytes of the intestinal epithelium, and are responsible for the production and storage of about 95% of 5-HT in the body [2].

5-HT released can act on the intrinsic nerves and vagal endings. It has diverse functions in regulating visceral sensitivity, gastrointestinal motility, emotion, pain, appetite, sensory perception, cognition, sexual activity, sleep etc [3]. These functions are mainly associated with the metabolic kinetics of 5-HT in different tissues. Synthesis of 5-HT is modulated by the rate-limiting enzyme tryptophan hydroxylase [4], its storage and release were regulated by vesicular monoamine transporter 1 [5] and its degradation is mediated by monoamine oxidase-A [6]. All of these factors together influence the level of 5-HT in vivo. Now-a-days, due to the functions of 5-HT in regulating different functions in body deviations in which can cause different diseases as stated below, the research on the 5-HT modulations is gaining momentum [7].

Diseases such as functional gastrointestinal disorders (FGIDs), gastrointestinal (GI) disorders [8], irritable bowel syndrome (IBS) [9], depression [10], cardiovascular diseases [11] were found to be closely associated with the metabolism of 5-HT. The symptoms are frequently accompanied by deviations in the levels of 5-HT or its receptors. Identification of levels of 5-HT in clinical samples became an important indicator for the diagnosis of diseases and/or identification of treatment outcomes. Many drugs such as alosetron, tegaserod, citalopram [12], paroxetine, venlafaxine [13], and prucalopride [14] have been developed based upon the metabolic kinetics of 5-HT.

Till date, the methods existing for the measurement of 5-HT in tissue, blood, and urine include LC–MS [15–17], ELISA [18] and electrochemical techniques [19, 20] coupled with high-speed chronoamperometry [21] or high performance liquid chromatography [22, 23]. However, these methods are costly, time-consuming and weren’t found to be either economic or convenient for screening of 5-HT in cell lines on a routine basis.

Besides, under the background that tryptophan metabolism/5-HT is increasingly popular in vitro study, the cell models of EC cells, such as QGP-1 [24] and RIN-14B [25, 26], in which marker genes of EC cells are highly expressed and 5-HT is secreted, were developed and a lot of in vitro studies are being carried out in laboratories [27]. EC cells can synthesize, secrete and even store 5-HT in it as a pool [28]. Finding out how the drugs or other factors, if any would influence the function of EC cells, would be greatly facilitated by the availability of simple assay method for the determination of 5-HT in cell lines and cell culture medium. The existing methods such as ELISA, LC–MS and other electro-chemical detection methods are expensive and complex techniques, and not suitable for use in routine basis. So we are attempting to improve the existing methods by replacing the expensive and complex detectors with convenient, economic HPLC–UV detector, and verifying this method in the matrix of RIN-14B cell extract and cell culture medium which makes it the preferred choice for routine use in laboratories.

## Experimental

### Cell line, chemicals, and reagents

About 95% 5-HT in the body is synthesized by EC cells [2], RIN-14B cell line which expresses EC cell marker genes [25, 26] is the most common model cell line to study EC cells in vitro and so we choose it as our preferred cell line for the identification of 5-HT in vitro. It was obtained from American Type Culture Collection (ATCC; CRL-2059) and was recovered, cultured and passaged following the instructions of ATCC. RPMI medium 1640, which is the medium ATCC recommend for RIN-14B cell line culture, was purchased from Gibco, Life technologies (NY, USA). Serotonin hydrochloride and tryptophan were purchased from Sigma Aldrich (USA), Methanol and acetonitrile (HPLC grade) were from Tedia Company, Inc., Fairfield (USA). Distilled water was prepared using a Millipore Milli-Q purification system (Millipore, Billerica, MA). KH_2_PO_4_ which was obtained from Sinopharm Chemical Reagent Co. Ltd., was used in the preparation of an aqueous solution of KH_2_PO_4_ (0.05 mol/L).

### Apparatus and HPLC–UV conditions

A high speed centrifuge (Thermo Fisher Scientific Inc. USA) was used to centrifuge the medium and cell samples. Analytical balance (Mettler-Toledo, Switzerland), the Agilent HPLC instrument (Agilent Technologies, USA) equipped with an on-line Degasser Agilent 1260 Infinity, Agilent 1260 Bin pump, 1260 ALS automatic sample introduction system and an Agilent 1290 Thermostat temperature controller was used for the HPLC experiment. For detection of 5-HT, the equipment was connected to an Agilent 1260 DAD VL UV diode array detector. We actualized the chromatographic separation of 5-HT with an Agilent Zorbax Extend C18 Column (4.6 × 250 mm, 5 μm) under isocratic elution. The mobile phase is 0.05 mol/L KH_2_PO_4_ (apparent pH = 5)/acetonitrile (90:10, V/V), and the wavelength of the UV detector was set at 280 nm.

### Preparation of stock solutions and quality control samples

To prepare a 100 μg/mL serotonin hydrochloride as the stock solution, serotonin hydrochloride sample powder (1.02 mg) was added to 10 mL of RPMI medium 1640 (FBS free) and pH was adjusted to 7 as per manufacturer’s instructions. The stock solution was then diluted subsequently into 9 calibrators with concentrations of 10 μg/mL, 5 μg/mL, 2.5 μg/mL, 1.25 μg/mL, 0.8 μg/mL, 0.4 μg/mL, 0.2 μg/mL, 0.1 μg/mL and 0.05 μg/mL respectively. Each solution is prepared in 3 mL volume and all solutions were preserved at 4 °C.

Quality control (QC) samples of 5-HT at low, middle and high concentrations (0.3, 4 and 7.5 μg/mL of serotonin hydrochloride respectively) were prepared independently and preserved at 4 °C.

Further, in consideration of tryptophan (TRP) which is one of the nutritional ingredients in 1640 medium, a 5 μg of TRP in 1 mL of 0.05 mol/L KH_2_PO_4_ standard solution was prepared for qualitative analysis of tryptophan in the cell culture medium and stored at 4 °C.

### Sample preparation procedure

**Fig. 2:**
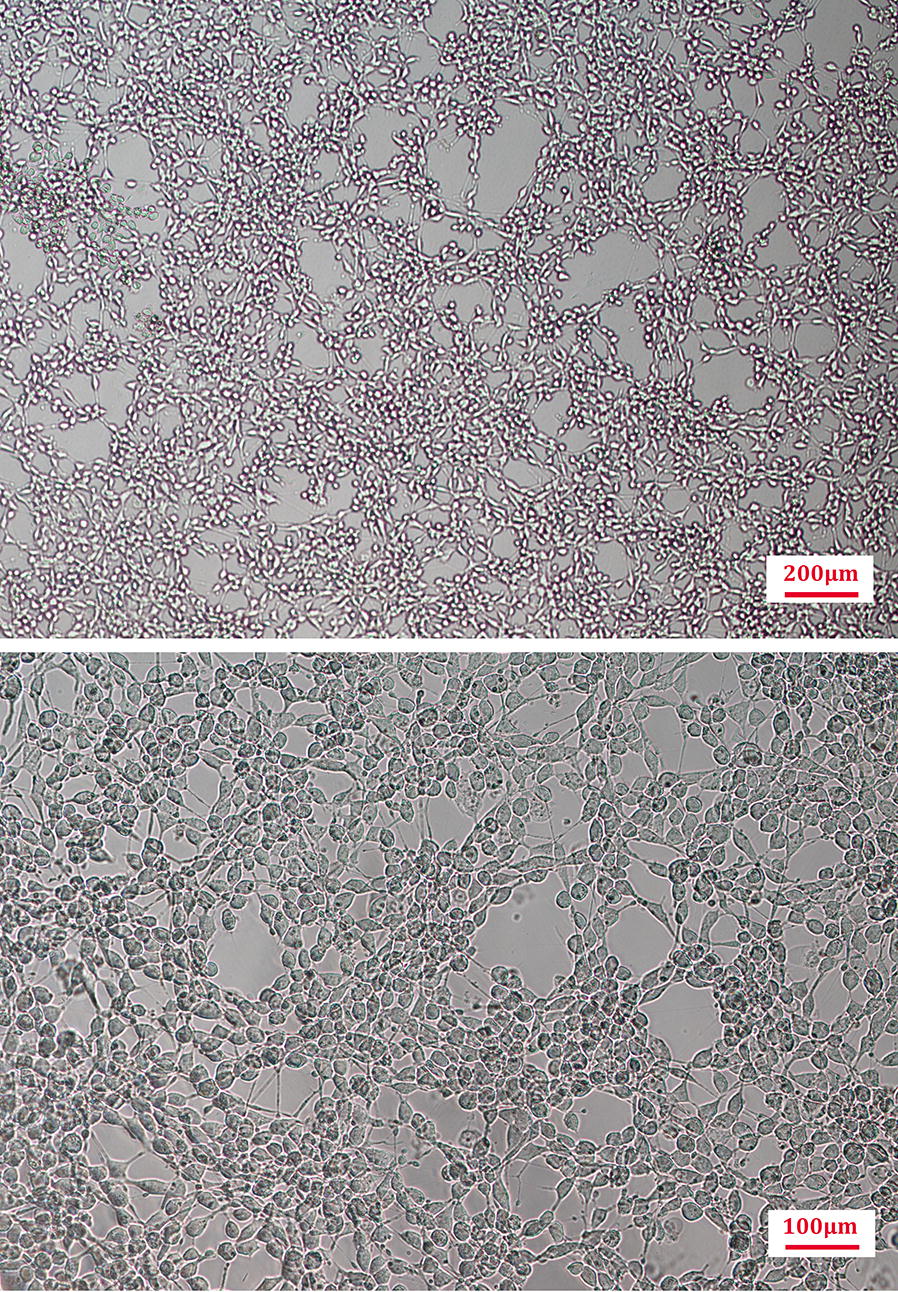
Representative imagines of RIN-14B cells (cultured for 48 h, Scale bar 200 μm/100 μm)

RIN-14B cell suspension was inoculated into dishes with 10 mL of RPMI medium 1640 containing 10% (V/V) fetal bovine serum (FBS), made the concentration about 1.5 × 10^5^ cells/mL, and cultivated for 20 h for adherence. The medium then was replaced with FBS free RPMI medium 1640 and cultured for 2 days (Fig. 2) to achieve the performance characteristics suitable for studies on EC cells and relationships between 5-HT and drugs [26, 29]. Using a pipette, 1.2 mL of culture medium was drawn from a cell culture dish, transferred into 1.5 mL EP tube and centrifuge at 14,000 rpm for 20 min at 4 °C. 1 mL of supernatant was taken into the sample vials for HPLC detection. Cell culture medium (CM) samples were preserved at 4 °C.

**Fig. 3:**
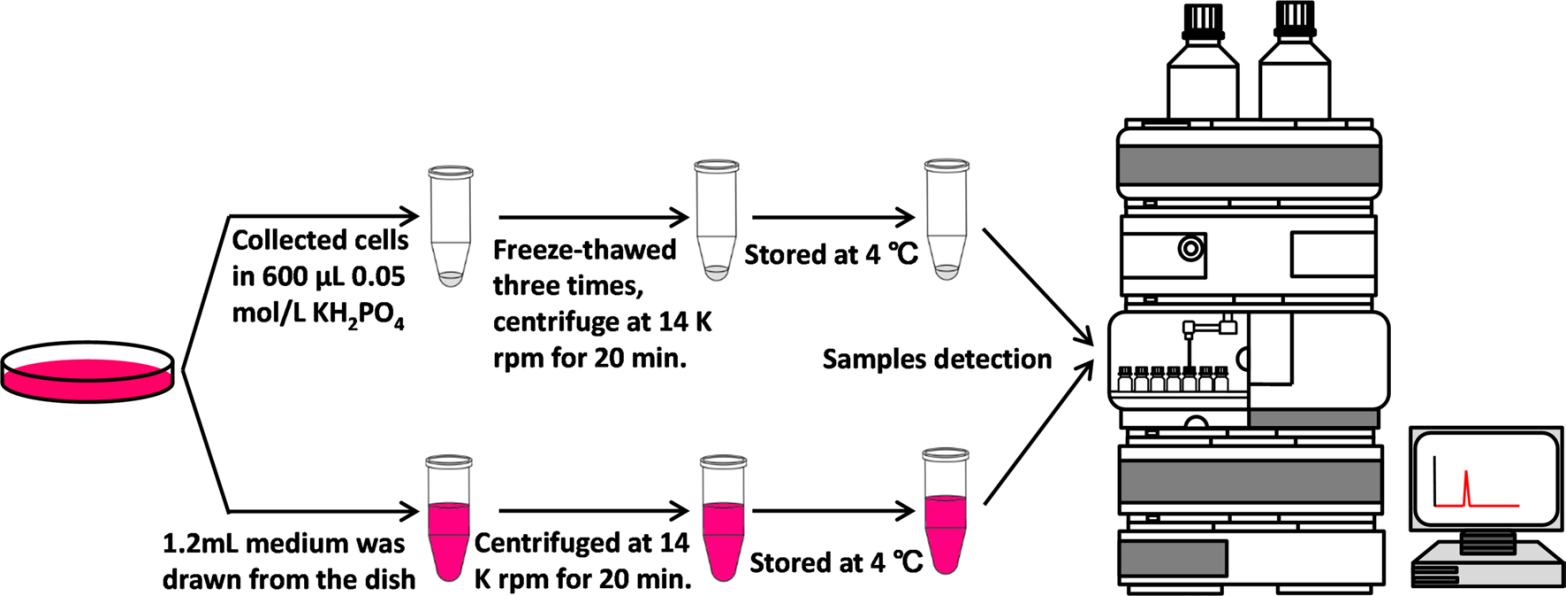
Flow chart of the experiment

The medium was removed from the dishes, cleaned twice with PBS (pH = 7.0) preheated at 37 °C to dispose the disturbance from 5-HT in the medium. To each dish 600 μL of 0.05 mol/L KH_2_PO_4_ (apparent pH = 5) pre-cooled to 4 °C was added and the cells were scraped from the bottom of the dish with cell scrapers. The scraped cells from each culture dish were collected into a 1.5 mL EP tube, freeze-thawed three times to break cells and release the 5-HT, then centrifuged at 14,000 rpm for 20 min at 4 °C. 300 μL of supernatant was taken into the sample vials for HPLC detection (Fig. 3). Cell extract (CE) samples were preserved in a refrigerator at 4 °C.

## Method validation and statistical analysis

Selectivity, accuracy, precision, linearity stability and residual analysis were determined according to Chinese pharmacopeia [30] guidelines, a brief account is as follows.

Selectivity of the method for 5-HT was estimated by detecting possible interference exists in 1640 medium. Three FBS-free 1640 medium samples with varying concentrations of 5-HT (0.04, 0.2, 1.0 μg/mL) were analyzed.

Accuracy and precision were assessed using QC samples which spiked with serotonin hydrochloride at three different concentrations, 0.3, 4 and 7.5 μg/mL, we tested replicates (n = 5) of QC samples and calculated as the unknown samples. To determine intra- and inter-day accuracy and precision, QC samples were measured in replicates (n = 5) on the same day and different days, accuracy was calculated by comparison of mean assay results (n = 5 for each QC sample) with the nominal concentrations (Table 1), with acceptability limits set at ± 10%. The intra-and inter-day precision was indicated as coefficients of variation (CV), an acceptability limit was set at ≤ 10%.

**Table 1:** Intra-and inter-day assay precision and accuracy for the determination of 5-HT in RPMI medium 1640

ASSAY	Nominal concentration (μg/mL)	Mean measured concentration (μg/mL)	CV (%)	Accuracy (%)
Intra‐assay (n = 5)	0.1	0.093	2.45	92.96
	0.3	0.305	1.12	101.71
	4	4.060	1.25	101.50
	7.5	7.493	0.32	99.90
Inter‐assay (n = 15)	0.1	0.092	4.63	92.02
	0.3	0.310	3.82	103.28
	4	3.981	4.69	99.53
	7.5	7.518	0.62	100.24

Intra-and inter-day assay precision and accuracy for the determination of 5-HT in RPMI medium 1640

Linearity was expressed by assaying on 9 separate calibration samples with concentrations 10 μg/mL, 5 μg/mL, 2.5 μg/mL, 1.25 μg/mL, 0.8 μg/mL, 0.4 μg/mL, 0.2 μg/mL, 0.1 μg/mL and 0.05 μg/mL respectively. Calibration curves were drawn by plotting the serotonin hydrochloride peak area against respective concentration of each calibrator, the linearity of the plots was tested with linear regression analysis.

Back-calculation of serotonin hydrochloride level in each calibration standard was performed, and presented with CV and accuracy (Table 2).

**Table 2:** Back-calculated concentrations of calibration standards

Back calculation (n = 3)	Nominal concentration (μg/mL)							
	0.1	0.2	0.4	0.8	1.25	2.5	5	10
Mean	0.105	0.209	0.413	0.808	1.272	2.540	5.086	10.137
SD	0.004	0.008	0.010	0.018	0.019	0.039	0.075	0.186
CV (%)	3.44	4.01	2.45	2.20	1.46	1.54	1.48	1.85
Accuracy (%)	104.94	104.70	103.30	100.97	101.77	101.60	101.71	101.37

Here we defined the limit of detection (LOD) as the lowest serotonin hydrochloride concentration which could generate chromatographic peak with a signal-to-noise ratio > 3, it was assessed after serial dilutions of the lower calibrator. The limit of quantitation (LOQ) was collected as the lowest concentration of the calibration range, and validated its precision was < 15% (CV) and accuracy within ± 15%, respectively (n = 5).

To investigate the stability characteristic of samples, serotonin hydrochloride in RPMI medium 1640 at concentrations of 0.3 and 7.5 μg/mL were exposed to three different conditions, storage at room temperature for 24 h, three freeze–thaw cycles and storage at 4 °C for 60 days. Assay results were obtained after exposure to these conditions were compared with the results of fresh samples (Table 3).

**Table 3:** 5-HT stability in spiked medium samples (n = 5)

Storage condition	Nominal concentration (μg/mL)	Mean measured concentration (μg/mL) ± SD	95% confidence intervals	Accuracy (%)
Freshly prepared samples	0.3	0.305 ± 0.003	0.301–0.309	101.70
	7.5	7.494 ± 0.024	7.463–7.522	99.92
24 h at room temperature	0.3	0.299 ± 0.004	0.295–0.304	99.80
	7.5	7.514 ± 0.022	7.487–7.542	100.19
Three freeze–thaw cycles	0.3	0.297 ± 0.003	0.294–0.300	99.03
	7.5	7.516 ± 0.011	7.502–7.530	100.21
60 days at 4 ℃	0.3	0.310 ± 0.001	0.309–0.311	103.03
	7.5	7.518 ± 0.004	7.512–7.524	100.24

Data were acquired and analyzed with the Agilent LC1260 software. Statistical significance was set at P ≤ 0.05. Statistical analyses were performed using GraphPad Prism (GraphPad Prism 5.00, San Diego, CA, USA).

## Results

### Method development

The UV–Vis spectra data were collected from 200 to 800 nm to pick the optimal wavelengths for measurements, and 280 nm was found to be the optimal wavelength to identify 5-HT.

To find out the best mobile phase polarity and resolution, we adjusted proportion of 0.05 mol/L potassium dihydrogen phosphate (KH_2_PO_4_) and acetonitrile, from 85:15 (v/v) to 98:2 (v/v), and retention time was advanced when polarity increasing, 90:10 (v/v) have the medium retention time and ensured better resolution.

The column oven temperature was set at 25 °C, and the injection volume was 60 μL.

In consideration of the low concentration of 5-HT in the CM samples, some CM samples were evaporated to dryness at 45 °C with a SAVANT SPD1010 concentrator (Thermo Fisher Scientific Inc. USA), the dry residue was dissolved in 0.05 mol/L KH_2_PO_4_ and filtered with Nylon Syringe Filter (Membrane Solutions, USA) for HPLC detection. But the results obtained weren’t found to be better than these from the CM sample and so this method was not used further. However, for CM samples in which 5-HT concentration is too low to detect can be used as an alternate method.

**Fig. 4:**
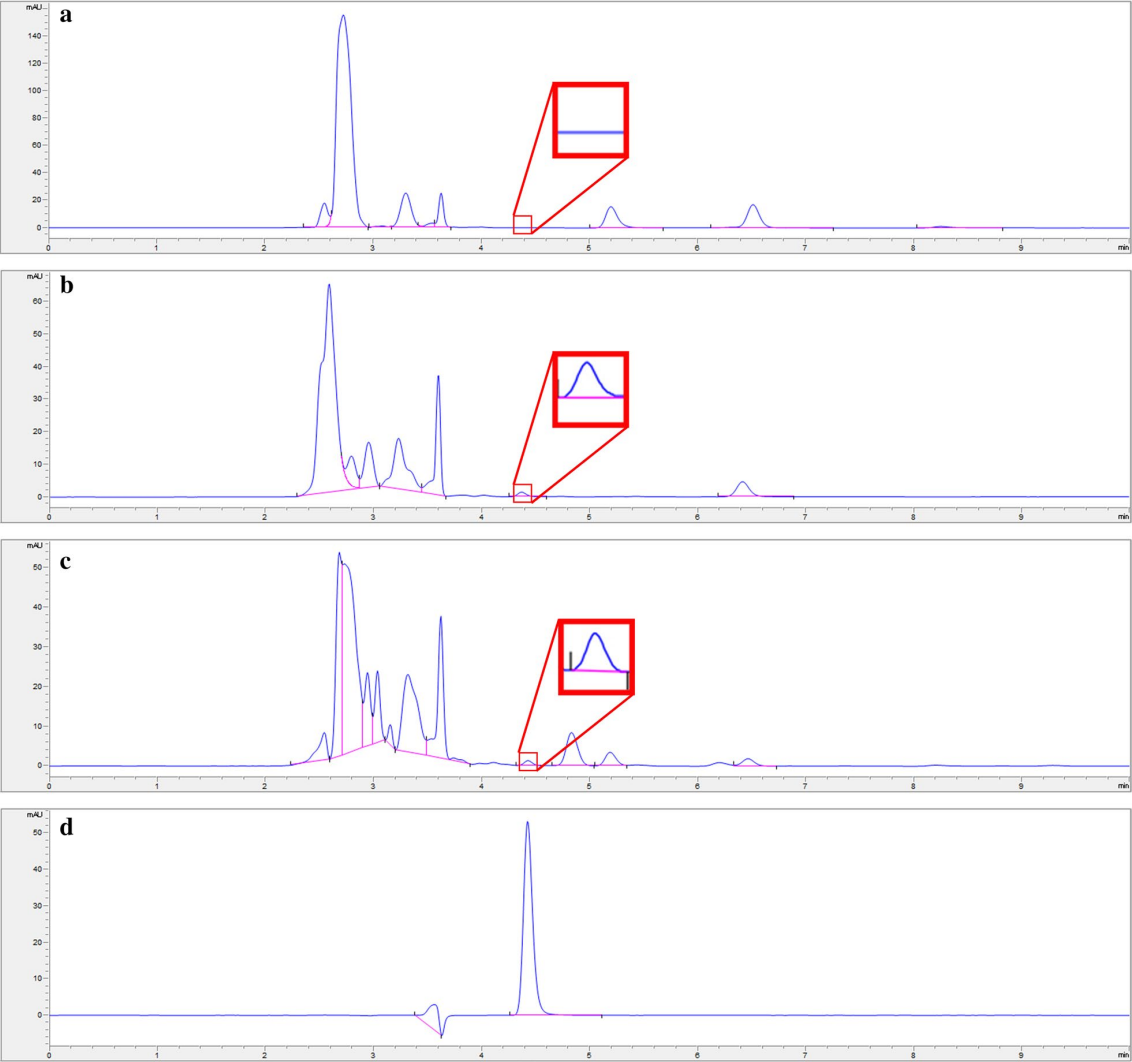
5-HT synthesis and secretion by RIN-14B cells. Chromatograms of FBS free 1640 medium sample (**a**), cell extract sample (**b**), FBS free 1640 medium sample in which RIN-14B were cultured for 24 h (**c**), and 5-HT standard sample (**d**)

**Fig. 5:**
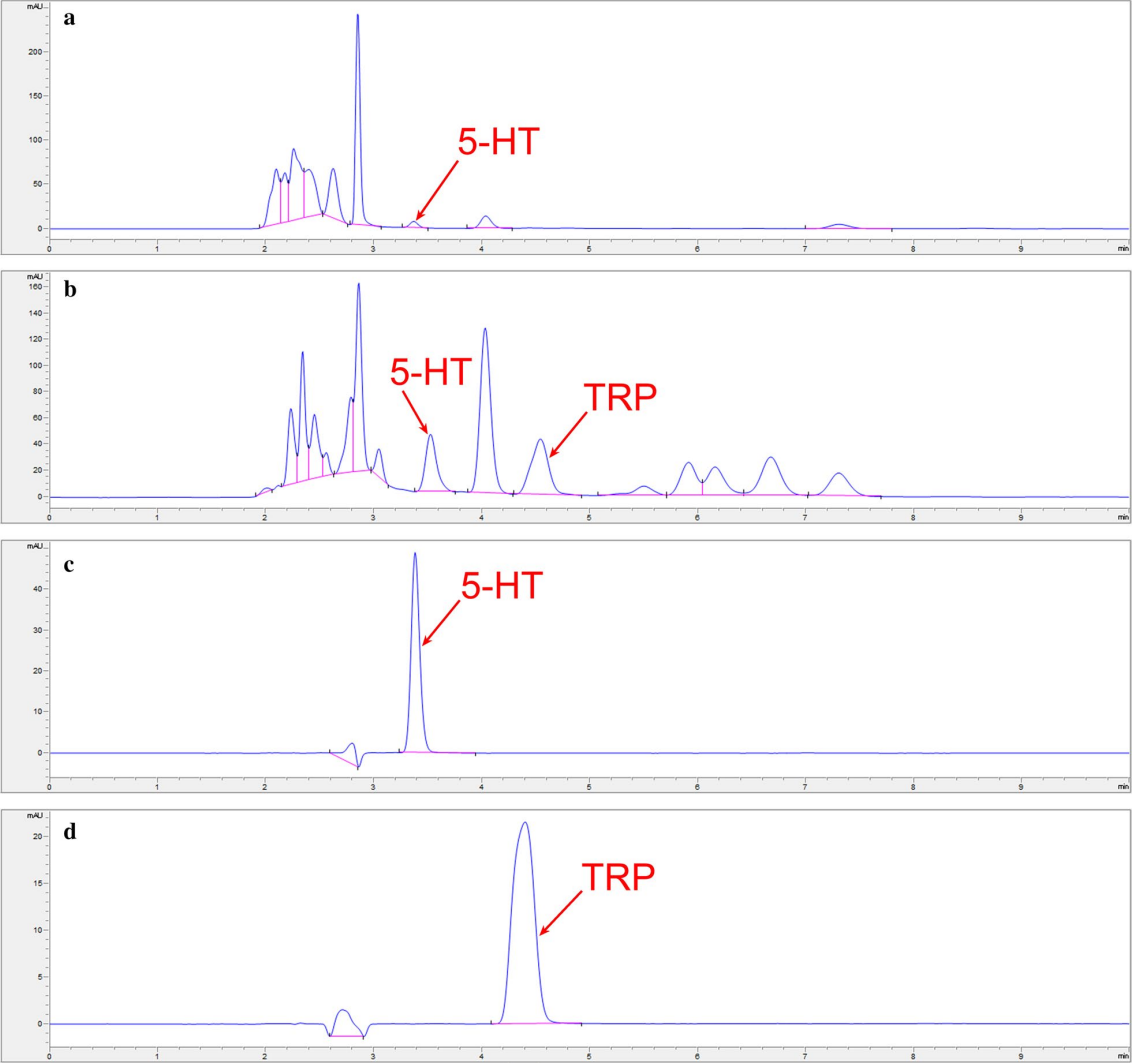
Representative chromatograms of a cell extracts sample (**a**), and cell culture medium sample (**b**), 5-HT standard sample (**c**), and TRP standard sample (**d**)

The physiological function of 5-HT synthesis and secretion of RIN-14B cells was verified (Fig. 4) firstly. Under the selected chromatographic conditions, 5-HT was eluted at about 3.5 min (Fig. 5a–c). The total duration of a chromatographic run required to obtain optimal resolution within 8 min (Fig. 5b).

Further, the standard solution of TRP (5 μg of TRP in 1 mL of 0.05 mol/L KH_2_PO_4_) was screened and was found to be eluted at about 4.5 min with good resolution ( > 3) (Fig. 5b, d).

### Selectivity

No interference was found has similar retention time to 5-HT in both CE and CM samples (Figs. 4c and 5b) suggesting that this method have good selectivity towards 5-HT in these samples.

### Accuracy and precision

Intra, inter-day accuracy and precision data are presented in Table 1. All of the data was met the criterion of acceptability recommended by the Chinese pharmacopeia guidelines, with overall intra, inter-day coefficient of variations (CVs) not exceeding 4.69%, and accuracy values between the range of 92.02–103.28%.

### Linearity and sensitivity

Calibration curves which were obtained using the calibration samples over the serotonin hydrochloride concentration range of 0.1–10 μg/mL, the results indicated that the concentration X of serotonin hydrochloride and the peak area Y shown a good linearity. The linearity equations were y = 84.56 x − 0.3408 (R^2^ = 0.9999). Limit of detection (LOD) and limit of quantitation (LOQ) values were 0.04 μg/mL and 0.1 μg/mL respectively. So the samples with 5-HT concentrations less than 0.1 μg/mL weren’t studied further. Accuracy and CV at LOQ for intra-day and inter-day were found to be 92.96%, 92.02%, 2.45% and 4.63% respectively (Table 1). The concentration of 5-HT in the above calibration standards were back-calculated. The accuracy and CV for the calibration range corresponding to LOQ (0.1 μg/mL) were found to be 104.94% and 3.44% respectively (Table 2).

### Residual analysis

**Fig. 6:**
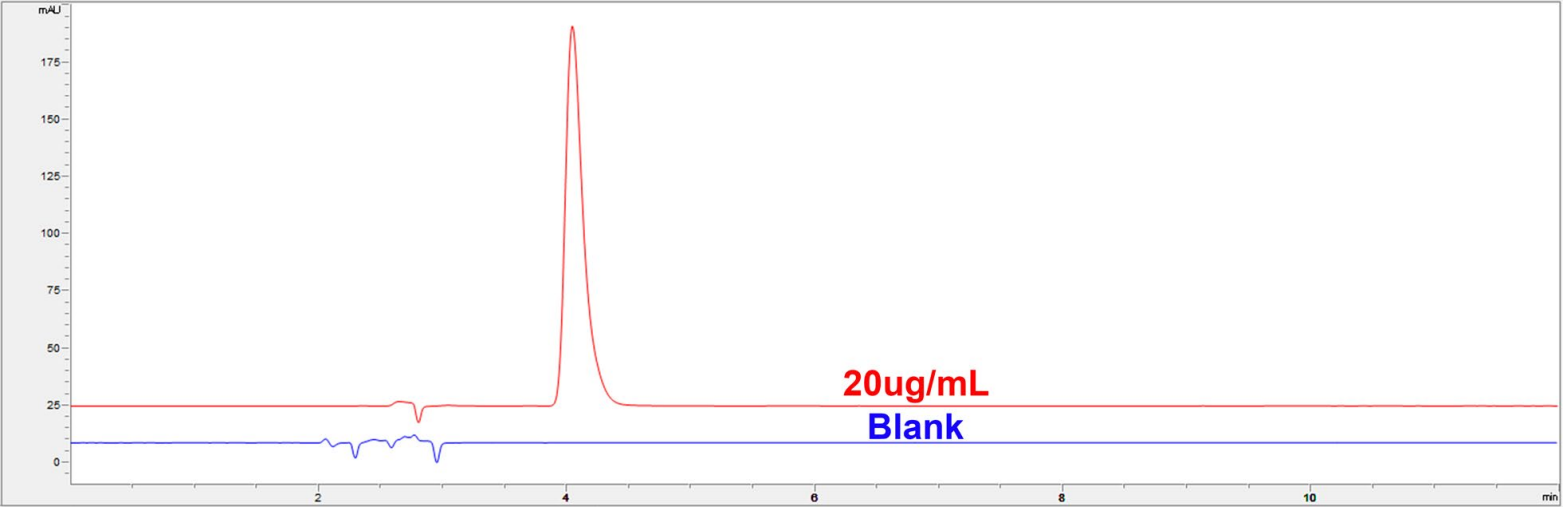
Superposition chromatograms of the 5-HT standard solution of 20 μg/mL and a blank sample

Residual analysis of the method so developed was carried out using a blank sample (0.05 mol/L KH_2_PO_4_ standard solution) and a 5-HT standard solution (20 μg of 5-HT in 1 mL of 0.05 mol/L KH_2_PO_4_ standard solution). 5-HT standard solution was run in the HPLC followed by the blank sample, no peak was detected in the blank sample at the retention time of 5-HT in the standard solution (Fig. 6).

### Stability

As shown in Table 3, no significant loss of serotonin was detected after samples were stored at room temperature for 24 h, over three freeze–thaw cycles or even after 60 days at 4 °C (repeated measures ANOVA, NS).

## Discussion

As mentioned earlier, the assays described to date for the determination of 5-HT in different biological samples use different methods or instruments [14–17], have substantial limitations in terms of applicability in laboratories where massive samples need to be analyzed within a short period of time. The present method is the first ever assay method for the determination of 5-HT in both the cells and the cell culture medium. This uses the HPLC–UV assay method have clear advantages in terms of speed, accuracy, and simplicity. In particular, the method provides a monolithic technology that permits to achieve short chromatographic run times (within 8 min) with the optimal solution, while ensures the accuracy and stability of detection.

The assay was tested for selectivity and found not to be subject to interference from other components in both the cell extract and cell culture medium, the 5-HT has a unique retention time under the selected chromatographic conditions (Fig. 5b). Another advantage is that the assay can be applied to measure the concentration of tryptophan (Fig. 5b, d), the precursor of 5-HT, upstream of anabolism metabolite, make it applicable to test the metabolic kinetics of 5-HT in cell lines.

Other performance characteristics of the assay are overall satisfactory. In particular, the sensitivity, with the LOQ as low as 0.1 μg/mL (Table 1), approaching the LOQ of HPLC–MS/MS method reported for detection of 5-HT in urine samples (470 nmol/L in the present study, VS 55.7 nmol/L in the literature method) [31], it is adequate to quantitate the concentrations of 5-HT in both cell extract and medium of RIN-14B. It is worth mentioning that the concentrations of 5-HT released into medium increased with the increase of time (Figs. 4c and 5b). Therefore, appropriate model making and drug delivery conditions in cell culture stage should be strictly controlled.

The retention time of 5-HT in the experiment slightly differs from RIN-14B cells physiological function verification experiment (Fig. 4), quantitative analysis of 5-HT in cell lines (Fig. 5) and residual analysis of 5-HT (Fig. 6). Several factors may contribute to the differences. especially due to the time gap between these experiments (60 days), several conditions such as frequent use during this time may cause aging of the chromatographic column, reagents, solvents, changes in column pressure may also influence the retention time of 5-HT. To eliminate the influence of these errors, a quality control sample with some concentration can be set and injected every 2 h is advised, and the column should be exclusively used in the experiment if possible.

## Conclusions

A simple, efficient and rapid HPLC–UV detection method for the determination of 5-HT in cell extract and cell culture medium was developed. The method was fully validated and can be considered to be suitable for researching the 5-HT secretion function of RIN-14B cells. It makes possible to study the physiological functions of EC cells in vitro, and this method may have potential applications for detecting other cell lines which have the function of secreting 5-HT. The present study provides a simple, accurate, economical and convenient method for these laboratories where LC–MS/MS is not available.

## Data Availability

The datasets generated and/or analyzed during the current study are not publicly available as this project isn’t finished yet and revealing the data at this point of time may affect the project. But the data is available from the corresponding author on reasonable request.

## Abbreviations

5-HT: 5-hydroxytryptamine
i.d.: internal diameter
EC: enterochromaffin
HPLC–UV: high performance liquid chromatography–ultraviolet detector
EE: enteroendocrine
GI: gastrointestinal
FGIDs: functional gastrointestinal disorders
ELISA: enzyme-linked immunosorbent assay
ATCC: American Type Culture Collection
FBS: fetal bovine serum
PBS: phosphate buffer solution
TRP: tryptophan
